# Biomarkers in the Diagnosis, Management, and Prognostication of Perioperative Right Ventricular Failure in Cardiac Surgery—Are We There Yet?

**DOI:** 10.3390/jcm8040559

**Published:** 2019-04-25

**Authors:** Habib Jabagi, Lisa M. Mielniczuk, Peter P. Liu, Marc Ruel, Louise Y. Sun

**Affiliations:** 1 Divisions of Cardiac Surgery, University of Ottawa Heart Institute, Ottawa, ON K1Y 4W7, Canada; hjabagi@ottawaheart.ca (H.J.); mruel@ottawaheart.ca (M.R.); 2 Cardiology, University of Ottawa Heart Institute, Ottawa, ON K1Y 4W7, Canada; LMielniczuk@ottawaheart.ca (L.M.M.); pliu@ottawaheart.ca (P.P.L.); 3 Cardiac Anesthesiology, University of Ottawa Heart Institute, Ottawa, ON K1Y 4W7, Canada; 4 School of Epidemiology and Public Health, University of Ottawa, Ottawa, ON K1G 5Z3, Canada

**Keywords:** right ventricular failure, biomarkers, cardiac surgery, natriuretic peptides, Galectin-3, ST2, sST2

## Abstract

Right ventricular failure (RVF) is a major risk factor for end organ morbidity and mortality following cardiac surgery. Perioperative RVF is difficult to predict and detect, and to date, no convenient, accurate, or reproducible measure of right ventricular (RV) function is available. Few studies have examined the use of biomarkers in RVF, and even fewer have examined their utility in the perioperative setting of patients undergoing cardiac surgery. Of the available classes of biomarkers, this review focuses on biomarkers of (1) inflammation and (2) myocyte injury/stress, due to their superior potential in perioperative RV assessment, including Galectin 3, ST2/sST2, CRP, cTN/hs-cTn, and BNP/NT-proBNP. This review was performed to help highlight the importance of perioperative RV function in patients undergoing cardiac surgery, to review the current modalities of RV assessment, and to provide a review of RV specific biomarkers and their potential utilization in the clinical and perioperative setting in cardiac surgery. Based on current evidence, we suggest the potential utility of ST2, sST2, Gal-3, CRP, hs-cTn, and NT-proBNP in predicting and detecting RVF in cardiac surgery patients, as they encompass the multifaceted nature of perioperative RVF and warrant further investigation to establish their clinical utility.

## 1. Introduction

Acute right ventricular failure (RVF) is a major risk factor for end organ morbidity and mortality following cardiac surgery. It is associated with difficult separation from cardiopulmonary bypass (CPB), renal and hepatic dysfunction, an up to 75% increase in in-hospital mortality, and poor late survival [1,2]. RVF is present in almost 50% of patients with postoperative hemodynamic instability [3] and is especially relevant in those who undergo surgery for congenital heart disease, mitral valve disease complicated by pulmonary hypertension (PH), coronary artery disease (CAD), orthotopic heart transplantation, and left ventricular (LV) assist device (LVAD) implantation. RVF is difficult to predict and diagnose in the perioperative setting [4]. This in turn may result in missed opportunities for optimization, prevention, and early treatment. Our review focuses on a novel aspect of RVF prediction, detection, and prognostication using biomarkers. We will begin with a brief review of the pathophysiology of perioperative RVF and traditional perioperative right ventricular (RV) assessment modalities, followed by the potential role of biomarkers in the perioperative assessment of RV function, risk stratification, and prognostication. Although biomarkers have yet to be established as clinical tools in the perioperative assessment of RV function, the current manuscript highlights their potential based on current evidence and provides recommendations for future research.

## 2. The Importance of RV Function Post Cardiac Surgery

Perioperative RVF is deadly. In a study of 52 patients requiring inotropic support following cardiac surgery, severe, isolated LV failure was associated with 33% (*n* = 3/10) in-hospital mortality, while severe, isolated RVF was associated with 90% (*n* = 9/10) mortality, and those with biventricular failure were associated with an 82% (*n* = 9/11) mortality rate, highlighting the importance of RV function [5]. In fact, RV dysfunction is an independent predictor of major adverse cardiac events such as cardiac death and HF hospitalization [6], and the addition of RV function improved risk stratification in those undergoing surgeries for CAD, congenital heart disease, and end stage HF [1]. Furthermore, in patients undergoing surgery for acute pulmonary embolism (PE), acute RVF is associated with higher rates of intraoperative cardiopulmonary resuscitation and mortality, as well as mortality at 30 days [7,8]. Despite its prevalence and prognostic importance, perioperative RVF remains poorly understood and investigated [4]. Existing RV studies are often limited by their retrospective design and small prospective sample sizes, as randomization is difficult due to its multi-faceted etiology [1]. The absence of best practice guidelines on the diagnosis and treatment of perioperative RVF is also concerning.

## 3. Etiology and Definition

The most common cause of acute RVF is LV failure. Other common perioperative etiologies are summarized in Table 1. RVF is defined physiologically as an inability of the RV to provide adequate blood flow through the pulmonary circulation at normal right atrial pressure (RAP) [9] and clinically by the presence of hypotension, an RAP >15 mmHg, and clear lungs [2]. Additional diagnostic criteria including pulmonary artery pressure and cardiac index have been used perioperatively [10]. However, these measures are confounded by the presence of LV systolic or diastolic dysfunction, raised intrathoracic pressure in mechanically ventilated patients, pre-existing PH, and tricuspid regurgitation [2]. To date, a widely applied definition of perioperative RVF is only available in the context of LVAD implantation, as postoperative need for inotropic and/or vasodilator support for ≥14 days, a right ventricular assist device (RVAD), or inhaled nitric oxide for >48 h [11]. Recently, a three-item criteria was proposed in non-LVAD patients, as (1) hemodynamic instability, defined as difficult or complex separation from CPB, (2) >20% reduction in RV fraction area change as measured by two-dimensional echocardiography, and (3) anatomical visualization of impaired or absent RV wall motion, by direct intraoperative visual inspection [12].

## 4. Traditional Approaches to Measuring RV Function

Perioperative assessment of RV function is complicated by a relative lack of practical and reproducible modalities in this setting [13] as well as the interplay between intrinsic biventricular myocardial performance and dynamic RV loading conditions [14]. Due to relative under-investigation of RV function compared to the LV [4,5,14], practical and reproducible means of measurement do not exist [13]. Table 2 summarizes common perioperative RV assessment approaches, their indications, advantages, and disadvantages.

Cardiac magnetic resonance imaging (MRI) is regarded as the current gold standard for non-invasive RV assessment [15], and right heart catheterization using pulmonary artery catheters is the invasive gold standard [13,16]. Widespread use of cardiac MRI is limited by its higher cost, longer procedural time, and the impracticality of MRI-specific safety precautions in mechanically ventilated patients and those with metal implants or temporary pacing wires. The routine use of pulmonary artery catheters is dwindling in many centers, in favor of less invasive methods such as central venous pressure (CVP). However, CVP measurements are influenced by mechanical ventilation, respiratory pathology, and changes in preload [17].

Echocardiography is readily available and frequently used perioperatively to assess RV function. The acquisition of perioperative transthoracic images (TTE) is challenged by the presence of positive pressure ventilation, chest tubes, bandages, and the fact that established quantitative measures of RV function are only validated in TTEs of spontaneously breathing patients [18]. Meanwhile, the widely used two-dimensional transesophageal echocardiography (TEE) is limited by the RV’s complex geometry [19]. Additional limitations of echocardiography are its subjectivity to inter- and intra-observer variability [20] and the fact that visual estimates of RV function often do not correlate well with the actual degree of venous congestion and end organ function.

## 5. Biomarkers and Perioperative RV Function

The limitations in current RV assessment modalities highlight the need for reliable, non-invasive, and cost-effective measures in the perioperative setting. With the advent of precision medicine, there is rising interest in the discovery of biomarkers to fulfill this role [16]. According to the World Health Organization, a biomarker is defined as any substance, structure, or process that can be measured in the body or its products and that influences or predicts the incidence of outcome or disease [27]. Most perioperative biomarker studies have used natriuretic peptides to quantify and predict LV failure [28], and perioperative research on RV biomarkers is lacking [29]. HF biomarkers are generally classified into markers of inflammation, myocyte injury and stress, neurohormonal activation, and extracellular matrix (ECM) turnover. In order for a biomarker to be clinically useful, it must be measurable, provide new information, and be able to assist in clinical decision-making (Table 3) [30]. This review will focus on the first two classes of biomarkers, as they have greater practical potential in the perioperative setting.

### 5.1. Biomarkers of Inflammation

Tissue injury initiates an inflammatory response in which proinflammatory cytokines are released into the bloodstream to repair damaged tissues [31]. The following inflammatory biomarkers are potentially relevant to the perioperative setting.

#### 5.1.1. Suppressor of Tumorgenicity 2 (ST2) and Soluble ST2 (sST2)

ST2 is a member of the IL-1 receptor family and exists in both insoluble membrane-bound and soluble forms (sST2). The binding of ST2 to IL-33 (a mediator of inflammatory disease that is also produced during processes of myocyte stress, hypertrophy, and fibrosis) [31] confers cardioprotection by reducing fibrosis and hypertrophy, ultimately preserving ventricular function [32]. This cardioprotective effect is reversed by the binding of IL-33 to sST2 [31].

High serum levels of sST2 differentiated between HF and non-cardiac etiologies of shortness of breath [33]. In addition, sST2 level weakly correlated with NYHA functional class (*r* = 0.13), LVEF (*r* = 0.13), creatinine clearance (*r* = 0.22), BNP (*r* = 0.29), NT-proBNP (*r* = 0.41), and C-reactive protein (*r* = 0.43; *p* < 0.05), one year after acute HF presentation [34]. In patients with acute decompensated HF, a sST2 cutoff value of >0.49 ng/mL had 72% sensitivity, 56% specificity, and 39% positive and 84% negative predictive values for predicting 1-year mortality [34,35]. When a cutoff value of >0.2 ng/mL was used, the negative predictive value for 1-year mortality increased to 96% [35]. In contrast, ST2 >35 ng/mL was associated with significantly increased risk of all-cause death or hospitalization (HR, 1.48; *p* < 0.0001), CV death, or HF hospitalization (HR, 2.14; *p* < 0.0001), and all-cause mortality (HR, 2.33; *p* < 0.0001) over the 32-month follow-up [36]. Elevated sST2 also predicts death and HF onset within 30 days following acute myocardial infarction (MI) [37,38,39].

Although circulating levels of sST2 have been implicated in the outcomes of patients with LV failure [36], recent studies suggest these levels may reflect responses of the RV to congestion [40,41]. Zheng et al. found correlations between sST2 levels and PH disease severity and clinical deterioration [42]. Expanding on these findings, Broch et al. examined patients with isolated arrhythmogenic RV cardiomyopathy and found an association between sST2 and RV function and arrhythmia load [43]. Ojji et al. also found correlations between sST2 and RV systolic pressure, diastolic diameter, and right atrial area in a cohort of hypertensive HF patients [44].

In summary, ST2 and sST2 are potential biomarkers of RV remodeling and may have a role in predicting short- and long-term mortality in those undergoing cardiac surgery [30]. Being unaffected by age, BMI, atrial fibrillation, HF, the specific cause of cardiomyopathy (i.e., ischemic vs. non-ischemic), and analytical interference by bilirubin, hemoglobin, triglycerides, cholesterol, or total protein [34], these biomarkers may be more specific than natriuretic peptides in detecting RVF and should be investigated in the perioperative setting.

#### 5.1.2. Galectin 3 (Gal-3)

Gal-3 is a macrophage product member of the lectins family, which is a group of proteins that interact specifically with carbohydrate sugars beta-galactosides [16,30]. Galectins play an important role in immune and inflammatory responses [45], wound repair [46], and pathways that regulate cardiac contractility and inflammatory cascades following injury [16]. Gal-3 is abundant in the spleen, lung, stomach, colon, and uterus, while normally expressed in lower amounts in the kidneys, pancreas, liver, and heart [47]. Significant increases in Gal-3 levels in the latter organs are correlated with pathophysiological states.

Cardiac remodeling is an essential feature of HF and is closely linked to disease progression [30]. Fibroblasts and macrophages are responsible for the initiation and progression of tissue fibrosis [48], and the expression of Gal-3 is increased in activated macrophages, which stimulates the maladaptive pathological cardiac responses such as remodeling, fibroblast proliferation, and collagen deposition [49]. Sharma et al. first described the Gal-3 gene expression in rat HF models and observed considerable collagen deposition with pericardial installation of Gal-3 [49]. The PRIDE study was the first to measure Gal-3 in humans [50], noting higher levels in patients with HF than those without (median 9.2 ng/mL vs. 6.9 ng/mL, *p* < 0.001). A study comparing NT-proBNP and Gal-3 in diagnosing acute HF demonstrated that, while NT-proBNP afforded more diagnostic accuracy for HF (with an area under curve (AUC) of 0.94 for NT-proBNP, *p* < 0.0002, and an AUC of 0.72 for Gal-3, *p* < 0.0001), Gal-3 was the strongest predictor of 60-day mortality (OR 10.3, 95% CI 1.6–174.1, *p* < 0.01) or a combination of death/recurrent HF within 60 days (OR 14.3, 95% CI 5.6–45, *p* < 0.001) [51]. In addition, the combined elevation of Gal-3 and NT-proBNP was a stronger predictor of mortality than either alone (*p* < 0.05) [51,52]. Gruson et al. showed that Gal-3 levels correlated with LV failure severity [53]. In addition, a cutoff of >19.2 ng/mL predicted long-term cardiovascular death, provided incremental prognostic information to BNP, and was a cost-effective means of reducing the length of hospital stay [53].

Milting et al. highlighted the possible utility of plasma Gal-3 as a biomarker of RV function in patients with advanced HF requiring LVAD support. In this study, the presence of LVAD reduced plasma BNP, deeming it impractical in prognostication. However, elevated plasma Gal-3 were observed in patients who died compared to those who survived to transplantation [30]. These findings were corroborated by de Boer et al., who found Gal-3 to be an independent predictor of adverse outcomes (all-cause mortality and HF hospitalization) in HF patients (HR 1.38, 95% CI 1.07–1.78, *p* = 0.015), especially those with preserved LVEF [52].

Gal-3 also correlates with RV function in the context of isolated PH. PH leads to both morphological and functional changes to the RV including contractile function, chamber size, hypertrophy, and changes in the ECM [54], all of which occur in the absence of LV failure. Fenster et al. found that serum Gal-3 levels were significantly elevated in PH subjects than controls and that Gal-3 correlated with RVEF (*r* = −0.44, *p* = 0.03), end diastolic volume index (*r* = 0.42, *p* = 0.03), end systolic volume index (*r* = 0.44, *p* = 0.027), mass index (*r* = 0.47, *p* = 0.016), systolic pressure (*r* = 0.55, *p* < 0.001), and strain (*r* = 0.43, *p* = 0.03) [54].

The potential utility of Gal-3 has not been studied in the perioperative context. In addition to being a marker of LV failure, Gal-3 has clearly been shown to correlate with RV function in the absence of LV failure, making it a promising RV biomarker for cardiac surgery patients. Possible areas of investigation include risk stratification and prognostication in patients receiving LVADs, as well as in PH patients requiring cardiac surgery.

#### 5.1.3. C-Reactive Protein (CRP)

CRP is a non-specific acute phase reactant that is predominantly produced by the liver and released into the bloodstream within minutes of inflammatory processes such as tissue injury and infection [55]. Although nonspecific, CRP has been used to assess patients with HF [56], PH [57], and PE [58]. In a study of PH patients, Quarck et al. showed correlations between CRP and NYHA class (*r* = 0.23), RAP (*r* = 0.25), and 6-min walking distance (*r* = −0.19). In addition, CRP was found to be significantly higher in non-survivors than in survivors (*p* = 0.003) [57]. These authors concluded that CRP has a role in predicting outcomes and response to therapy in PH patients. CRP has also been linked to survival in the setting of acute PE, with higher levels being observed in those with RV dysfunction and death [58]. The positive correlation between CRP, RAP, and the degree of RV dysfunction points to its possible utility as a prognostic marker in the settings of PH and acute PE [57,58]. This notion is further supported by findings of the large Multi-Ethnic Study of Atherosclerosis Right Ventricle Study, where elevated CRP may be linked to negative RV structural changes [56,59]. In summary, serum CRP is a rapid, readily available assay that may have practical value in the assessment of perioperative RV function and prognostication.

### 5.2. Biomarkers of Myocyte Injury and Stretch

#### 5.2.1. Cardiac Troponins

Cardiac troponins (cTn) have traditionally been used in the diagnosis of MI but may also be elevated in the setting of HF [60]. In the setting of acute PE, elevated cTn predicted RV hypokinesis and mortality [61,62] and correlated more strongly with future risk of HF than ischemic events [63]. Optimal cutoff value for predicting RV hypokinesis in this context is 0.1 ng/mL [62,64]. The major drawback of cTn as a RV biomarker is its lack of specificity, especially in the presence of chronic stable CAD or concomitant renal disease.

#### 5.2.2. High Sensitivity Troponin (hs-cTn)

Hs-cTn is a more sensitive predictor of mortality and readmissions in both stable and acute decompensated HF [60,65]. The utility of this assay in detecting RVF has only been explored in small studies in non-operative settings. Specifically, elevated hs-cTn levels are associated with RV dysfunction in patients with acute PE [66]. In addition, in the setting of PH, hsTn levels increase specifically in proportion to the severity of RV dysfunction [67]. In patients with previous Tetrology of Fallot repair, hs-cTn increases specifically with increasing RV volume and worsening RVEF [68]. In the setting of coronary artery bypass grafting (CAPG), elevated hs-cTn is associated with postoperative MI, the need for vasopressor support, prolonged mechanical ventilation, an increased intensive care unit (ICU) length of stay, and mortality [69]. The predictive and diagnostic utility of hs-cTn should be investigated in patients undergoing cardiac surgery, as it may provide information on postoperative recovery.

#### 5.2.3. Natriuretic Peptides

To date, only BNP and its precursor NT-proBNP have been validated for clinical use in HF patients [70]. Current HF guidelines recommend monitoring of BNP or NT-proBNP levels not only for diagnosis, but also for monitoring disease progression and prognosis [30]. However, conditions such as age, sex, renal function, body mass index, and anemia confound the observed BNP and NT-proBNP values [71].

BNP is transcribed and produced in ventricular myocytes, [72] in response to shear stress and myocardial stretch in pressure or volume overloaded states [16]. It counteracts maladaptive responses to volume homeostasis and peripheral vascular resistance by inducing natriuresis, diuresis, and vasodilation [73]. BNP production is normally about 10 pg/mL in healthy individuals [74]. Under conditions of increased myocardial stretch, the pre-proBNP is upregulated and cleaved into the more stable, biologically active BNP and NT-proBNP [16]. In patients presenting to the emergency department with acute dyspnea, elevated BNP differentiated HF from other causes of dyspnea (AUC 0.91), and BNP levels correlated directly with HF severity [75].

NT-proBNP is a better diagnostic test than BNP due to its longer half-life (70 min vs. 20 min) and thus higher plasma concentrations [16]. It has a greater negative predictive value for acute HF compared to BNP and has an optimal cutoff value of 300 pg/mL [75]. NT-proBNP is also superior to BNP as a predictor of survival in PH patients due to its ability to reflect the degree of hemodynamic impairment while being unaffected by the presence of renal insufficiency, which is a beneficial feature in the perioperative setting [76].

In the acute cardiac surgical setting, preoperative BNP is associated with early outcomes in terms of: ICU length of stay, duration of postoperative mechanical ventilation, and inotropic support [77]. In fact, several studies found BNP and NT-proBNP to be better predictors of nonfatal cardiac events, ICU length of stay, and all-cause in-hospital mortality following cardiac surgery than the well-established EuroSCORE [78,79]. The addition of NT-proBNP to EuroSCORE II also improved the clinician’s ability to predict severe circulatory failure following isolated CABG for ACS [80].

RV dysfunction has been implicated in reduced exercise capacity [81] and cardiovascular death [82] in both idiopathic and ischemic cardiomyopathy [15]. Natriuretic peptides are elevated specifically in patients with RV dysfunction in a variety of clinical settings. In the setting of PE and preserved LV systolic function, BNP was elevated specifically in those who developed RV dysfunction [83]. A meta-analysis in the setting of acute PE also found BNP and NT-proBNP to be elevated in patients with RV dysfunction, and that these biomarkers predicted complicated in-hospital course (OR 6.8, 95% CI 4.4–10) and 30-day mortality (OR 7.6, 95% CI 3.4–17) [84]. In the setting of PH, BNP, and NT-proBNP increase specifically in proportion to the degree of RV distension and wall stress, thus providing important prognostic information [15,85,86]. Thus, higher BNP levels are independently correlated with mortality, RV pressure or volume overload, and reduced RV EF in this setting [15,31,86]. In HF patients with comparable LVEF, Passino et al. found higher BNP levels in the presence of RV volume overload and dysfunction and concluded that there was an inverse relationship between RVEF and natriuretic peptides levels [15].

NT-proBNP provides a simple and effective snapshot of RV cellular function and correlates well with the gold standard cardiac MRI [85]. Its role in predicting and detecting RVF warrants further exploration in the full scope of cardiac surgery practice, especially in light of widely adopted perioperative measures of RV function and volume status such as TEE and CVP.

#### 5.2.4. ANP

ANP has also been implicated in the diagnosis and prognosis of patients with RV dysfunction [15,86]. ANP is secreted by the atria in healthy individuals and by the LV in patients with LV dysfunction [87]. Like BNP, ANP is a counter-regulatory hormone that functions as vasodilator, natriuretic peptide, and inhibitor of the renin-angiotensin-aldosterone system [86]. Persistently elevated ANP levels were evident in HF refractory to treatment, while decreasing ANP trends were observed in those who responded to therapy [88]. Elevated ANP levels have previously been described in isolated RV dysfunction in the context of PH [89] and PE [90]. However, ANP is less sensitive and specific than BNP in detecting RVF, making it less desirable as a target for perioperative research [91].

## 6. Implications for Research and Clinical Practice

The incorporation of biomarkers in the perioperative management of cardiac surgery patients represents a promising strategy to improve patient care. To date, RV-specific biomarkers have not yet been identified in the perioperative setting [29], and this presents an opportunity for future research. We suggest that the potential utility of ST2, sST2, Gal-3, CRP, hs-cTn, and NT-proBNP in predicting and detecting RVF be explored and validated for use in conjunction with other diagnostic modalities such as echocardiography and RHC. The addition of biomarkers can provide objective measures of RV function, and help to overcome the limitations of existing modalities such as inter-observer variability in the visual estimate of RV function by echo and the invasiveness of RHC. This proposed multimodal approach can provide a rapid, inexpensive, and comprehensive snapshot of RV structure, function and the state of venous congestion (Table 4). In addition, serial biomarker measurements may assist in monitoring response to therapy, assessing whether a patient is optimized for surgery, and administering personalized interventions perioperatively and in long-term follow up.

Current data support the role of serum ST2, sST2, and Gal-3 as markers of RVF in the presence of preserved LVEF and/or adequate LV mechanical circulatory support (MCSP), and similar investigations need to be extended to the perioperative setting. For instance, ST2 may help to differentiate cardiovascular vs. non-cardiovascular causes of shortness of breath and hypoxemia in the postoperative patient and to predict and detect acute RVF and common RVF-associated complications and death. Gal-3 has already been shown to be highly reflective of RVF in cardiac surgery patients requiring MCSP [92,93], holding promise as a marker to help prioritize transplant listings and provide personalized, goal-directed perioperative management to prevent and treat RVF in high-risk patients. It is to be noted that these biomarkers are still in the investigative phase and are limited by the practicality of their assay kits. For instance, the traditional 96-well ELISA plates may not be run frequently, as enough samples would have to be gathered before each run. The advent of rapid point of care ST2, sST2 [94], and Gal-3 assays [53] brings the promise of a shortened processing time (20 min), thus allowing clinicians to react more promptly based on these test results.

CRP and hs-cTn are a routine part of many clinical practices. Both have rapid laboratory turnaround times, are relatively inexpensive to measure, and have the ability to predict mortality and acute RVF in at-risk non-surgical populations such as those with acute PE. These qualities raise the possibility of using these biomarkers to rapidly confirm suspected cases of acute RVF post-cardiac surgery, thus allowing timely interventions. Potential drawbacks such as a lack of specificity for RVF will also need to be investigated in the perioperative setting.

NT-proBNP has been shown to correlate with the gold standard cardiac MRI measurement of RV morphology and function. Serum NT-proBNP can be measured rapidly in the laboratory and by using a reliable and inexpensive point of care assay. Observational studies have already shown the utility of this biomarker in acute perioperative RVF, and in risk stratifying patients with circulatory failure post-CABG. Further studies need to focus on validation in a broader range of cardiac procedures and to determine whether serial perioperative NT-proBNP measurements would lead to improved outcomes and reduced healthcare cost.

## 7. Conclusions

Perioperative RVF is associated with increased mortality, morbidity, and healthcare cost [21]. Cardiac surgical patients are a susceptible group in whom objective, reproducible, and non-invasive diagnostic measures of RV function are needed. Biomarkers offer rapid, unbiased and non-invasive molecular level insight that may be used to inform personalized, etiology-specific therapy in the face of rapidly evolving perioperative physiology. Our review has identified several biomarkers whose role in the assessment and management RVF deserve further investigation in the perioperative setting. These biomarkers may prove to be useful in identifying high-risk patients for personalized, etiology-specific therapy, to monitor the state of preoperative optimization and perioperative response to therapy, as well as to supplement RHC and echocardiograms in establishing transplant priority and eligibility for LVAD therapy. A single biomarker measurement may alert clinicians of the need to initiate RV-specific therapy early, to prevent clinical deterioration and end organ failure. This may ultimately lead to shortened ICU and hospital lengths of stay, reduce the need for unplanned RV mechanical support, and improve patient outcomes while minimizing healthcare cost.

## Figures and Tables

**Table 1:** Common causes of right ventricular failure (RVF) post cardiac surgery.

Mechanism	Etiologies
Intrinsic RV failure (normal afterload)	Ischemia/infarction Coronary embolism (air or thrombus) Occlusive CAD Bypass graft dysfunction/thrombosis Postoperative RV dysfunction Suboptimal myocardial protection intraoperatively Inflammatory CPB effects (long CPB times) Arrhythmias (AVNRT or loss of AV synchrony) Cardiotomy
RV failure secondary to increased afterload	LVF MVD with PH Protamine induced PH Ischemia-reperfusion injury PE ARDS Pre-existing PH or OSA
RV failure secondary to increased volume overload	Excessive blood transfusions Excessive fluid administration Severe TR or PR
Cardiac anatomical abnormalities	CHD ASD VSD
Miscellaneous	OHT PH Prolonged donor ischemic time Obstruction of PA anastomotic site Acute rejection LVAD Acute LV unloading with institution of LVAD support Sepsis

Abbreviations: RV—right ventricular; CAD—coronary artery disease; CHD—congenital heart disease; CPB—cardiopulmonary bypass; AVNRT—atrioventricular nodal re-entrant tachycardia; AV—atrioventricular; PH—pulmonary hypertension; RVOT—right ventricular outflow obstruction; OHT—orthotopic heart transplant; LVAD—left ventricular assist device; LVF—left ventricular failure; MVD- mitral valve disease; OSA—obstructive sleep apnea; PA—pulmonary artery; PR—pulmonary regurgitation; TR—tricuspid regurgitation; ASD—atrial septal defect; VSD—ventricular septal defect; ARDS—acute respiratory distress syndrome; PE—pulmonary embolism.

**Table 2:** Current perioperative right ventricular (RV) assessment modalities.

Diagnostic Tool	Indication	Information Provided	Current Perioperative Use in Cardiac Surgery	Role in Perioperative Risk Stratification of RVF in Cardiac Surgery Patients
Cardiac MRI	Cardiomyopathies Myocarditis Hemochromatosis patients Congenital heart diseases Coronary artery disease	Gold standard for quantitative noninvasive measurement of RV volume, mass and ejection fraction [21] Myocardial ischemia and viability (delayed gadolinium enhancement of fibrotic/scarred myocardium) General structure and function	Surgical planning in complex congenital cases Pre-CABG to determine revascularization strategy	Not feasible: Expensive Time consuming Postoperative assessment not logical, especially in ventilated and/or ICU patients Repeat scans for continuous RVF assessment impossible
RHC	Identify or exclude HF Shock (differentiate cardiac vs. non-cardiac) Congenital heart disease Cardiomyopathy PH (differentiate primary vs. secondary) Heart transplant	Gold standard for invasive assessment of the RV Cardiac output Chamber pressures Pulmonary artery pressure Ejection fraction Vascular resistances	Pre-transplant: assessment of PH Post-transplant: myocardial biopsy for signs of rejection and for assessment of heart function Intraoperative assessment of heart function and responses to inotropic and vasopressive medications Assessment of valve abnormalities Diagnosis of cardiac tamponade Assessment of cardiac shunts	High diagnostic yield: Continuous monitoring of RV function Help differentiate right vs. left HF Provides information on responses of the RV to treatment Not routinely done for all cardiac surgical patients
Chest X-ray	Pulmonary assessment Fluid overload	RV enlargement (globular appearance of the cardiac silhouette and loss of the retrosternal airspace on lateral films) [21] Enlarged main PA in patients with PH [22]	Routinely done as part of preoperative assessment	Low diagnostic yield
RAP (or CVP)	All patients undergoing cardiac surgery with a central line in place	Volume status Indirect measure of RV function	Routinely placed in all cardiac surgery patients RV prognostication in patients with LVAD	Medium diagnostic yield: Indirect measure of RV function RAP > 15 mmHg (RVF after LVAD) [23] RAP:PCWP > 0.63 (RVF after LVAD) [24] RAP:PCWP > 0.86 (RVF after acute MI) [25] PVR > 3.6 WU (RVF after LVAD) [26]
ECHO	Heart failure CHD PAH Valvular disease Infective endocarditis Pericarditis Tamponade	General structure and function Ejection fraction Valve function and defects Cardiac output	Case dependent Assessment of valve repair/replacement Failure to wean patient from CPB	High diagnostic yield: Relatively easy to perform Transthoracic (noninvasive) and transesophageal (semi-invasive) methods available Direct RV function assessment (TAPSE) Not routinely done for all cardiac patients
Natriuretic Peptides (BNP and NT-proBNP)	Heart failure Acute PE PH Heart transplant	LV dysfunction RV dysfunction Prognostic information in patients with PH or PE	Pre-transplant patients	Identifying high-risk patients for personalized, etiology-specific therapy Deferral of non-emergency surgery for decongestive and vasodilator therapy Monitor response to therapy Identify high-risk patients for difficult CPB separation Prioritize patients on transplant waitlists Identify high-risk candidates for enrolment in clinical trials
Serum Markers (transaminases)	Heart failure	Differentiate between chronic and acute HF	Nil	Nil

Abbreviations: MRI—magnetic resonance imagining; RV—right ventricle; CABG—coronary artery bypass grafting; ICU—intensive care unit; RVF—right ventricular failure; RHC—right heart catheterization; HF—heart failure; PH—pulmonary artery hypertension; PA—pulmonary artery; RAP—right atrial pressure; CVP—central venous pressure; LVAD—left ventricular assist device; PCWP—pulmonary capillary wedge pressure; PVR—pulmonary vascular resistance; WU—wood unit; ECHO—echocardiography; CHD—congenital heart disease; CPB—cardiopulmonary bypass; TAPSE—tricuspid annular plane systolic excursion; BNP—brain natriuretic peptide; NT-proBNP—N-terminal pro-brain natriuretic peptide.

**Table 3:** Properties of a clinically useful biomarker.

Criteria	Details
Is the biomarker measurable?	Accurate and reproducible methods Rapid turnaround time Reasonable cost
Does the biomarker provide new information?	Strong and consistent association between biomarker and outcome or disease in multiple studies Provides information that is not available from clinical assessments and adds or improves upon existing tests
Will the biomarker assist clinical decisions making?	Superior to other available tests Assists decision-making and enhances clinical care

**Table 4:** RV specific biomarkers.

Biomarker	RV Specific Uses	Non-RV Uses
Biomarkers of Inflammation		
ST2 and sST2	Correlation with PH disease severity and clinical deterioration Increased levels associated with isolated arrhythmogenic RV cardiomyopathy Significant correlations between soluble sST2 and RV systolic pressure, diastolic diameter, and right atrial area have been shown Yet to be studied in RV prognostication in cardiac surgery patients	Differentiate dyspnea caused by HF vs. noncardiovascular etiologies Prognostic value in aiding risk stratification of heart failure patients at high risk of mortality and rehospitalization HF prediction post ACS
Galectin 3	Biomarker for RV function in patients on LVAD support Gal-3 is well correlated with RV function in the context of isolated PH	Diagnosing acute HF and assessing severity of HF based on NYHA Strong predictor of mortality and recurrent HF
CRP	High levels are associated with RV dysfunctional and mortality in acute PE Systemic inflammation as measured by CRP has been shown to specifically contribute to negative RV structural changes	Used to asses patients with HF, PH, and PE
Biomarkers of Myocyte Injury & Stress		
High Sensitivity Troponins	Elevated levels associated with RV dysfunction in acute PE Correlated with worsening RVEF post TOF repair Yet to be studied in the cardiac surgery population	Diagnosing acute MI
BNP and NT-proBNP	Elevated in patients with RV dysfunction in the setting of PE and PH Elevated levels in patients with comparable LVEF in the presence of RV dysfunction Yet to be studied in RV prognostication in cardiac surgery patients	Diagnosing and monitoring disease progression in HF patients Predictors of length of stay, inotropic support, and postoperative mechanical ventilation support in acute cardiac surgical setting

Abbreviations: PH—pulmonary artery hypertension; HF—heart failure; RV—right ventricular; ACS—acute coronary syndrome; LVAD—left ventricular assist device; CRP—C reactive protein; NYHA—new york heart association; PE—pulmonary embolism; BNP—brain natriuretic protein; MI—myocardial infarction; NT-proBNP—N-terminal pro-brain natriuretic peptide; LVEF—left ventricular ejection fraction.

## Abbreviations

**Variable**	**Definition**
RV	right ventricular
RVF	right ventricular failure
HF	heart failure
CVP	central venous pressure
RAP	right atrial pressure
CPB	cardiopulmonary bypass
PH	pulmonary hypertension
PE	pulmonary embolism
VAD (LVAD/RVAD)	ventricular assist device (left/right)
BNP/NT-pro-BNP	brain natriuretic peptide, N-terminal pro-brain natriuretic peptide
